# Supplementary material for: Comparison of Methods for Incorporating Related Data When Developing Clinical Prediction Models: A Simulation Study
Source: Biom J. 2026 Aug 27;68(5):e70166. doi: 10.1002/bimj.70166 (PMC13520826; doi:10.1002/bimj.70166)
Supplement: Supplementary file 2 [file BIMJ-68-e70166-s001.zip › third submission code review - R2/reproducibility_supplement/results/Plots/combined_plots/numpredictors_plot_combination_combined_combination_SC_ID8_TS0.25_DS4.pdf]

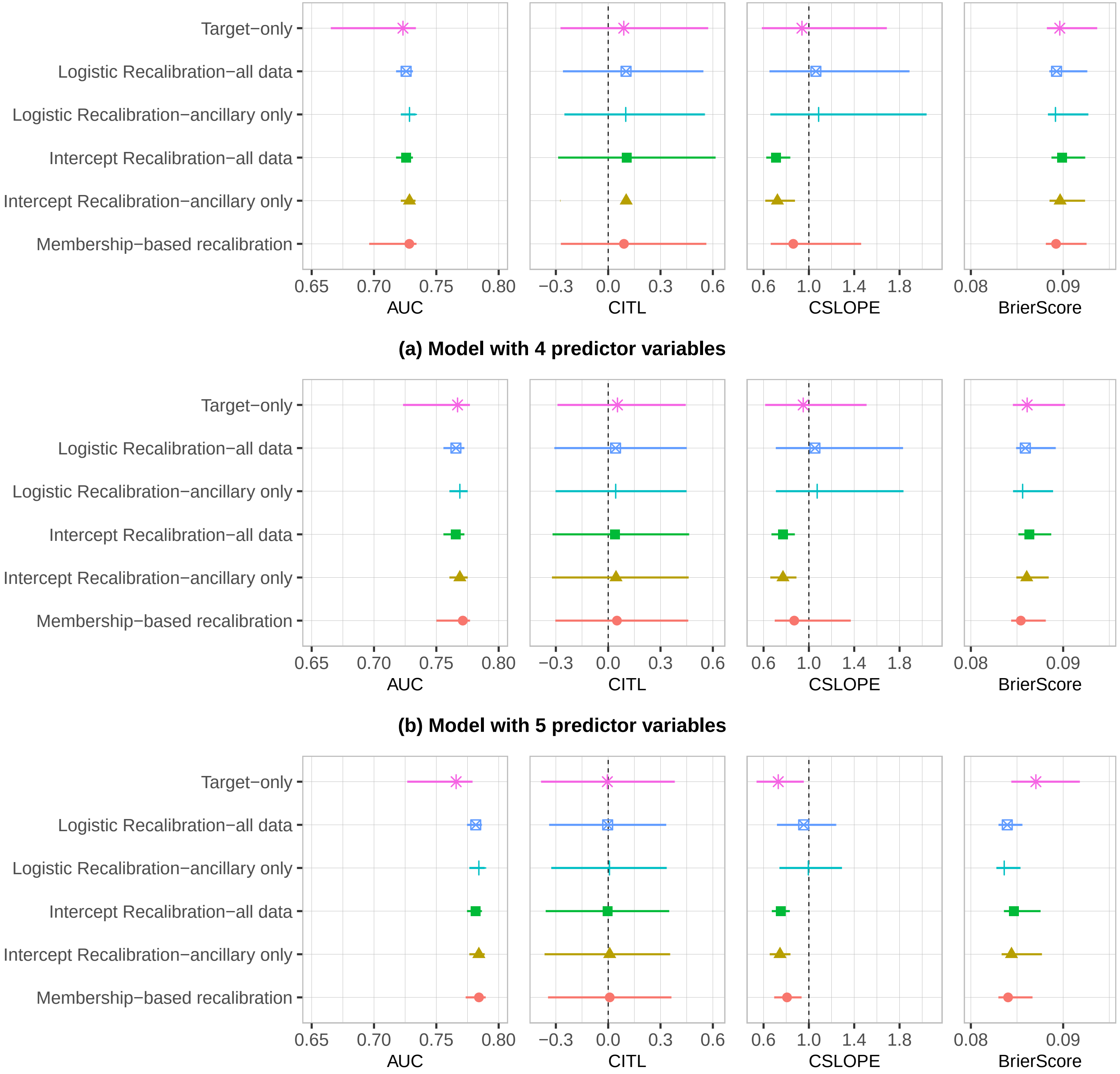


**(a) Model with 4 predictor variables**

**(b) Model with 5 predictor variables**

**(c) Model with 15 predictor variables**
